# Poly-γ-Glutamic Acid (PGA)-Producing *Bacillus* Species Isolated from *Kinema*, Indian Fermented Soybean Food

**DOI:** 10.3389/fmicb.2016.00971

**Published:** 2016-06-21

**Authors:** Rajen Chettri, Meera O. Bhutia, Jyoti P. Tamang

**Affiliations:** Department of Microbiology, School of Life Sciences, Sikkim University, GangtokIndia

**Keywords:** *Kinema*, *Bacillus*, fermented soybean, poly-glutamic acid

## Abstract

*Kinema*, an ethnic fermented, non-salted and sticky soybean food is consumed in the eastern part of India. The stickiness is one of the best qualities of good *kinema* preferred by consumers, which is due to the production of poly-γ-glutamic acid (PGA). Average load of *Bacillus* in *kinema* was 10^7^ cfu/g and of lactic acid bacteria was 10^3^ cfu/g. *Bacillus* spp. were screened for PGA-production and isolates of lactic acid bacteria were also tested for degradation of PGA. Only *Bacillus* produced PGA, none of lactic acid bacteria produced PGA. PGA-producing *Bacillus* spp. were identified by phenotypic characterization and also by 16S rRNA gene sequencing as *Bacillus subtilis, B. licheniformis* and *B. sonorensis.*

## Introduction

Poly-γ-polyglutamic acid (PGA), an amino acid polymer, is not synthesized by ribosomal proteins (Oppermann-Sanio and Steinbüchel, 2002); but is synthesized by Gram-positive bacteria (Yao et al., 2009) and few Gram-negative bacteria (Candela et al., 2009) produced as a polymer outside of the cell (Moraes et al., 2013). PGA-producing bacteria are mainly *Bacillus subtilis, B. anthracis, B. licheniformis, B. thuringensis, B. cereus, B. pumilus, B. amyloliquefaciens, B. mojavensis, B. atrophaeus, B. megaterium, Staphylococcus epidermidis, Natrialba aegyptiaca, Lysinibacillus sphaericus*, and *Fusobacterium nucleatum* (Kambourova et al., 2001; Cachat et al., 2008; Meerak et al., 2008; Candela et al., 2009; Cao et al., 2011). PGA is one of the functional properties of microorganisms present in fermented soybean foods (Tamang et al., 2016a). PGA is an anionic, biodegradable, water-soluble, non-toxic, and edible (Yoon et al., 2000; Zhang et al., 2011). Structurally there are two types of PGA: γ-PGA and α-PGA, which are composed of glutamic acids joined by γ or α linkages, respectively (Goto and Kunioka, 1992). γ-PGA has a structure of 5,000–10,000 units of D- and L-glutamic acids that generate a highly viscous solution when it accumulates in the culture medium (Ashiuchi et al., 2001; Tanimoto et al., 2001). PGA produced by *Bacillu*s spp. has potential applications as thickener, cryoprotectant, humectant, drug carrier, biological adhesive, heavy metal absorbent, etc., with biodegradability in the fields of food, cosmetics, medicine, and water treatments (Bajaj and Singhal, 2011; Ogunleye et al., 2015).

Ethnic people of North East India consume spontaneously fermented soybean foods as side dish in meals, which include *kinema*, *tungrymbai*, *hawaijar*, *bekang*, *aakhone*, and *peruyaan* (Tamang, 2015). *Kinema* is a naturally fermented, sticky, mild-ammoniacal flavor and non-salted soybean food of Sikkim and Darjeeling in India, east Nepal and west Bhutan. It is similar to *natto* of Japan, and *chungkokjang* of Korea. PGA is produced by *Bacillus* spp. in many Asian fermented soybean products giving the characteristic of a sticky texture to the product (Urushibata et al., 2002; Nishito et al., 2010) such as *natto* of Japan (Nagai, 2012; Kada et al., 2013), *chungkokjang* of Korea (Lee et al., 2010), *tungrymbai* and *bekang* of India (Chettri and Tamang, 2014), and *thau nao* of Thailand (Chunhachart et al., 2006). One of the criteria for good quality of *kinema* is high stickiness of the product preferred by consumers (Tamang and Nikkuni, 1996). Relative viscosity and stickiness are probably due to production of PGA by *Bacillus* spp. (Nagai et al., 1994; Tamang and Nikkuni, 1996). *B. subtilis* KK3:B4, isolated from naturally fermented *kinema* of India, produced high amount of relative viscosity of 20.1 (Tamang and Nikkuni, 1996). PGA-producing *Bacillus* strain was isolated from *kinema* of Nepal (Hara et al., 1995). Though several species of *Bacillus* such as *B. subtilis, B. licheniformis, B. cereus, B. circulans, B. thuringiensis*, and *B. sphaericus* were previously isolated from *kinema* using phenotypic characterization (Sarkar et al., 1994, 2002; Tamang, 2003; Tamang et al., 2016b); however, there has been no further report on PGA-producing strains/species of *Bacillus*, isolated from *kinema* samples of India. Hence we conducted this experiment. The present study was to screen PGA-producing species of *Bacillu*s from *kinema* and to identify species of *Bacillus* by 16S rRNA sequencing.

## Materials and Methods

### Sample Collection

Fresh samples of *kinema* were collected from different markets of Sikkim in India. Samples were collected aseptically in pre-sterile bottles, sealed, labeled, kept in an ice-box and were transported immediately to the laboratory. Samples were stored at 4°C for further microbial and biochemical analyses.

### Isolation of Microorganisms

Ten gram of sample was homogenized in 90 mL sterile physiological saline in a stomacher lab-blender (400, Seward, UK) for 1 min and a serial dilution was made. The diluents were heated at 100°C for 2 min for inactivation of vegetative cells of endospore bacteria (Tamang and Nikkuni, 1996), were isolated and enumerated on nutrient agar (MM012, HiMedia, India), and incubated for 24 h at 37°C. Lactic acid bacteria (LAB) were isolated on plates of MRS agar (M641, HiMedia, India) supplemented with 1% CaCO_3_ and incubated at 30°C in an anaerobic gas-jar (LE002, HiMedia, India) for 48–72 h. Total viable counts were determined on plate count agar (M091A, HiMedia, India) incubated at 30°C for 48–72 h. Isolated colonies were purified and were preserved in 15% (v/v) glycerol at -20°C for further analysis.

### Phenotypic Characterization

Cell morphology and motility of isolates were observed using a phase contrast microscope (Olympus CH3-BH-PC, Japan). Isolates were Gram-stained and tested for production of catalase, carbon dioxide from glucose, ammonia from arginine, growth at different temperatures, in different concentrations of NaCl and pH in nutrient broth (M002, HiMedia, India) following the method of Schillinger and Lücke (1987). Voges–Proskauer test, nitrate reduction, starch hydrolysis, casein hydrolysis, citrate utilization test, bile salt tolerance, anaerobic growth, and sugar fermentations were determined following the method of Duc et al. (2004). Taxonomic key of Slepecky and Hemphill (2006) was followed for identification of *Bacillus* spp.

### Measurement of Stickiness

Cultures were grown on phytone agar (Nagai et al., 1994) at 37°C for 24 h were pulled by touching with an inoculating needle and the stickiness was measured by the length of the thread using scale in cm.

### Screening of PGA

Screening of PGA by bacteria was done with a slightly modification of the method described by Nagai et al. (1997) and Meerak et al. (2007). *Bacillus* isolates were grown at 37°C for 24 h in a conical flask containing 100 ml of PGA medium that consisted of sodium glutamate 2.0%, glucose 2.0%, (NH_4_)_2_SO_4_ 1.0%, Na_2_HPO_4_ 0.1%, KH_2_PO_4_ 0.1%, MgSO_4_⋅7H_2_O 0.05%, Mn(Cl_2_)_4_.H_2_O 0.002%, FeCl_3_⋅7H_2_O 0.005% (Kunioka and Goto, 1994). The culture after incubation was centrifuged to obtain a supernatant that contained insoluble material. An equal volume of ethanol was added to the supernatant to get fibrous precipitate presumbly the PGA (Nagai et al., 1997).

Efficiency of PGA of the isolates were tested in different pH (5, 7.5, and 9) and temperature (30 and 45°C) following the method of Meerak et al. (2007).

### Degradation of PGA

Screening of LAB for degradation of PGA was performed following the method described by Tanaka et al. (1993). Strains were grown in MRS broth (M369, HiMedia, India), for 18-24 h at 30°C. The isolates were streaked on MRS agar plates containing 0.5% pure PGA (Sigma) solution (pH 4.5), and incubated at 30°C for 2–3 days. The plates were flooded with 5 ml of 18 N H_2_SO_4_ and allowed to stand for 30 min at room temperature. The presence of halo around the colony determines the degradation of PGA.

### Genomic DNA Isolation

Genomic DNA was isolated according to the method of Wilson (2001). Amplified 16S rDNA was obtained from each strain by polymerase chain reaction (PCR) with the universal primers; forward 5′-AGAGTTTGATCCTGGCTCAG-3′ and reverse 5′-AAGGAGGTGATCCAGCCGCA-3′ (Weisburg et al., 1991). The amplicons sizes ranged from 914 BP to 1814 BP.

### Gel Electrophoresis

The amplified DNA fragments were separated through gel electrophoresis by applying 10 μL of each PCR product with 1.5 μL of loading dye [(6×), DV4371, Promega, USA] into the wells of 1.5% agarose (V3125, Promega) gel containing 1.5 μL/mL ethidium bromide (H5041, Promega). DNA size markers (RMBD135, Genei; G5711, Promega) were added as standard for the calculation of size of the DNA fragments. The gel was run and photographed using gel documentation system (GelDoc FQ, Biorad, USA).

### 16S rDNA Sequence Analysis

The sequencing reactions were performed using ABI PRISM 3100 Genettic Analyzers (Applied Biosystems) in both direction with universal primers used for amplification. The electrophenogram data for 16S rDNA sequence was validated using Chromas 2.33 software.^1^ Sequences obtained were matched with previously published bacterial 16S rDNA sequences available in the GenBank database using BLAST and the Ribosomal Database Project (RDP).

### Phylogenetic Analysis

For phylogenetic analysis, 16S rDNA sequence of the isolates and reference sequence retrieved from NCBI-GenBank database were aligned with Clustal Omega. The resulting alignment were analysed with MEGA 6.0 to construct the phylogenetic tree. Phylogenetic tree was inferred with neighbor-joining (NJ) method (Saitou and Nei, 1987). Sequence divergence among the strain were quantified using Kimura-2-paramater distance model (Kimura, 1980). A total of 1,000 bootstrap replication were calculated for evaluation of the tree topology.

## Results and Discussion

### Phenotypic Identification

The average population of *Bacillus* spp. in *kinema* was 10^7^ cfu/g, LAB was 10^3^ cfu/g and total viable counts were 10 cfu/g, respectively (data not shown). Thirty-nine isolates of *Bacillu*s were isolated from 10 samples of *kinema*. Based on phenotypic characterization (data not shown) five species of *Bacillus* were identified from 10 samples of *kinema* as *B. subtilis, B. licheniformis, B. pumulis, B. sphaericus* and *B. cereus* (**Table 1**). About 90% of the total bacterial population found in *kinema* was *Bacillus*, indicating that *Bacillus* is the dominant bacterium in *kinema*. Sarkar and Tamang (1994) also reported that *Bacillus* is the predominant bacterium in *kinema*. *B. subtilis, B. licheniformis, B. cereus, B. circulans, B. thuringiensis*, and *B. sphaericus* were reported from *kinema* sample earlier (Sarkar et al., 1994, 2002; Nout et al., 1998; Tamang, 2003).

**Table 1:** Screening of stickiness, and PGA production at different pH and temperatures.

Organisms	Strain code	Stickiness (cm)	PGA production	
			pH 7.5	30°C
*Bacillus subtilis* (*n* = 13)	KAS:B5	16	++	**+++**
	KAS:B6	18	++	**+++**
	KAS:B18	6	+	+
	KAS:B29	16	++	**+++**
	KAS:B36	4	+	+
	KAS:B39	15	++	**+++**
	KLM:B68	3	+	+
	KLM:B78	3	+	+
	KLM:B86	4	+	+
	KLM:B98	4	+	+
	KAS:B102	20	++	**+++**
	KLM:B112	23	++	**+++**
	KLM:B114	2	+	+
*B. licheniformis* (*n* = 4)	KAS:B46	4	+	+
	KAS:B56	20	++	+++
	KLM:B92	21	++	+++
	KLM:B108	2	+	+
*B. pumulis* (*n* = 5)	KAS:B15	3	+	+
	KAS:B48	5	+	+
	KLM:B73	5	+	+
	KLM:B93	6	+	+
	KLM:B106	4	+	+
*B. sphaericus* (*n* = 8)	KAS:B9	2	+	+
	KAS:B16	4	+	+
	KAS:B19	5	+	+
	KAS:B49	6	+	+
	KLM:B66	3	+	+
	KLM:B72	2	+	+
	KLM:B82	2	+	+
	KLM:B96	2	+	+
*B. cereus* (*n* = 9)	KAS:B8	2	_	_
	KAS:B10	1	_	_
	KAS:B38	2	_	_
	KAS:B58	2	_	_
	KLM:B74	2	_	_
	KLM:B84	2	_	_
	KLM:B85	2	_	_
	KLM:B88	3	_	_
	KLM:B104	1	_	_

*n, number of isolates in parenthesis. +++, high clumping of insoluble precipitate; ++, more clumping of precipitate; +, moderate clumping of precipitate; -, no clumping of precipitate. No precipitate was observed in pH 5 and 9, and at 45°C.*

### Screening of PGA Production

**FIGURE 1:**
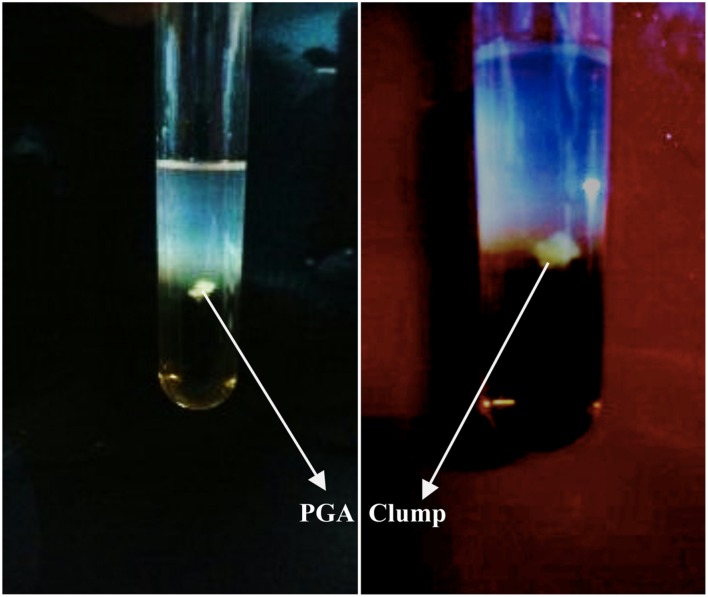
**Clumping of insoluble material presumably PGA biopolymer produced by *Bacillus subtilis* KAS:B5 after addition of ethanol into PGA medium**.

Stickiness of 39 isolates of *Bacillus* was measured (**Table 1**). The ability of 39 isolates of *Bacillus* were tested for production of PGA in PGA medium (Kunioka and Goto, 1994) in pH 5, 7.5, and 9, and at 30C and 45°C (**Table 1**). The isolates formed an insoluble material or fibrous precipitate after addition of equal volume of ethanol into the PGA medium (**Figure 1**) presumbly PGA biopolymer (Nagai et al., 1997; Ashiuchi et al., 2001). All species of *Bacillus* showed fibrous precipitate indicating the absence of PGA production except *B. cereus*.

We tested 25 isolates of LAB isolated from *kinema* for their ability to degrade poly-glutamic acid (PGA) to know whether LAB also produce PGA in *kinema* (data not shown). All LAB isolates were found to degrade PGA, indicating that they have no role in PGA production. Similar observations of degradation of PGA by LAB in fermented soybean were made earlier (Kimura and Fujimoto, 2010; Chettri and Tamang, 2014).

### Molecular Characterization

On the basis of high (+++) fibrous precipitate at 30^o^ C and pH 7.5, and stickiness of >15 cm (**Table 1**), 8 strains of *Bacillus* viz. KAS:B5, KAS:B29, KAS:B39, KAS:B56, KAS:B102, KAS:B6, KAS:B92, and KAS:B112 were selected and were identified by 16S rRNA sequencing. Based on the similarity search with blastN and EzTaxon server the strain KAS:B5 was identified as *B. subtilis*, KAS:B6 as *B. licheniformis*, KAS:B29 as *B. licheniformis*, KAS:B39 as *B. licheniformis*, KAS:B56 as *B. subtilis*, KAS:B92 as *B. licheniformis*, KAS:B102 as *B. licheniformis* and KAS:B112 as *B. sonorensis*. Recovery of *B. sonorensis* from *kinema* is the first report.

**FIGURE 2:**
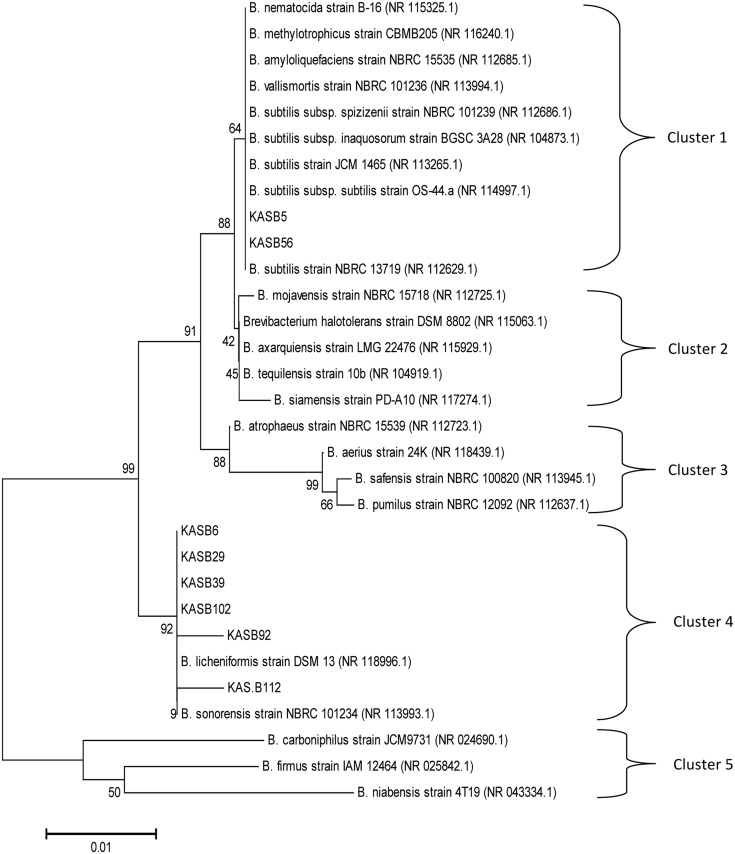
**Evolutionary relationships of the analyzed strains with their closest known taxa.** The evolutionary history was inferred using the Neighbor-Joining method. The tree was constructed based on the evolutionary distance calculated from 16S rRNA gene sequences using Kimura 2-parameter method. The percentage of replicate trees in which the associated taxa clustered together in the bootstrap test (1,000 replicates) are shown next to the branches. The tree is drawn to scale, with branch lengths in the same units as those of the evolutionary distances.

Phylogenetic tree was constructed with neighbor joining method based on the evolutionary distance calculated from 1,000 replicates has showed 5 distinct clusters (**Figure 2**), which were separated on a scale of 0.01 nucleotide substitution. The homogeny similarity of *Bacillu*s spp. and accession numbers were shown in **Table 2**. Out of 8 PGA-producing strains KAS:B5 and KAS:B56 showed similarities with *B. substilis* strain NBRC13719, *B. subtilis* subsp. *subtilis* strain OS44a and other strains of *subtilis* like JCM1465, NBRC 101236, NBRC 101239, and BGSC 3A28 with 64% of similarity percentage in cluster 1. KAS:B6, KAS:B29, KAS:B39, KAS:B102, and KAS:B92 were found in same clade of cluster 4 showing similarities with *B. licheniformis* DSM12 with 92% similarity and KAS:B112 showed similarities with *B. sonorensis* strain NBRC 101234 with 90% similarity. Strains KAS:B92 and KAS:B112 were found to show a distance gap between the other species of cluster 4 indicating the difference in nucleotide sequence and evolutionary lineage. In this paper, we could find that *B. subtilis* and *B. licheniformis* are PGA-producing bacteria in *kinema*. *B. subtilis* and *B. licheniformis* are the most widely used industrial producers of γ-PGA (Kambourova et al., 2001; Stanley and Lazazzera, 2005; Zhang et al., 2011).

**Table 2:** Homogeny of PGA-producing *Bacillus* isolated from *kinema*.

Strain code	*Bacillus*	Accession number	Homogeny (% similarity)
KAS:B5	*Bacillus subtilis*	KX262911	96
KAS:B6	*B. licheniformis*	KX262910	98
KAS:B29	*B. licheniformis*	KX261423	94
KAS:B39	*B. licheniformis*	KX261424	97
KAS:B56	*B. subtilis*	KX262912	97
KAS:B92	*B. licheniformis*	KX261426	97
KAS:B102	*B. licheniformis*	KX261425	96
KAS:B112	*B. sonorensis*	KX262913	97

## Conclusion

Consumers prefer slimy texture of *kinema* as good quality product. Presumably slimy material in fermented soybean food is polyglutamic acid, which has been reported from several Asian fermented foods produced by *Bacillus* spp. PGA, has several applications as foods as well as non-foods. The present study revealed that some species of *Bacillus* produced PGA in *kinem*a. Further investigation is needed to characterize and purify PGA produced by *Bacillus* spp. during natural fermentation of *kinem*a.

## Author Contributions

RC: screening of PGA-producing *Bacillus* from *kinema*, molecular identification of *Bacillus*, screening go PGA, stickiness, and preparation of draft paper. MOB: phenotypic identification. JPT: analysis of data, compilation and finalization of paper.

## Conflict of Interest Statement

The authors declare that the research was conducted in the absence of any commercial or financial relationships that could be construed as a potential conflict of interest.

## Footnotes

1 www.technelysium.com.au
